# Maternal gut microbiota and placenta-derived tissues microbes are important for initial gut microbial colonization in infants

**DOI:** 10.3389/fmicb.2025.1631590

**Published:** 2025-10-31

**Authors:** Ziyi Zhang, Longlong Jia, Bin Liu, Yanpin Liu, Junying Zhao, Yaling Wang, Minghui Zhang, Weicang Qiao, Baoyu Yang, Lingling Luo, Lijun Chen

**Affiliations:** ^1^Key Laboratory of Dairy Science, Ministry of Education, Food Science College, Northeast Agricultural University, Harbin, China; ^2^National Engineering Research Center of Dairy Health for Maternal and Child, Beijing Sanyuan Foods Co. Ltd., Beijing, China; ^3^Beijing Engineering Research Center of Dairy, Beijing Technical Innovation Center of Human Milk Research, Beijing Sanyuan Foods Co. Ltd., Beijing, China; ^4^College of Food Science and Engineering, Jilin Agricultural University, Changchun, Jilin, China; ^5^School of Food and Health, Beijing Technology and Business University, Beijing, China

**Keywords:** gut microbiota colonization, infant gut, maternal microbiota, vertical transmission, 16S rRNA

## Abstract

**Background:**

Early infant gut microbiota colonization, influenced by various factors, significantly affects future growth and development. However, results related to how the initial microbial ecology is established in the infant gut remain inconsistent.

**Results:**

In this study, we collected maternal and infant feces, vaginal secretions, placental tissues, breast milk, amniotic membrane tissues, umbilical cord blood, and breast skin for homology comparisons and for exploring the main sources of infant intestinal microbiota. Our results revealed that early infant gut microbiota originated mainly from the vertical transmission of maternal microbiota, and that vaginal microbiota did not affect infant gut microbiota colonization. Microbiota was detected in the placenta, supporting the idea that the uterus is not sterile. Moreover, we verified microbial composition-related similarities in the amniotic tissues and umbilical cord blood, further validating our hypothesis that gut microbiota in the early stages of infancy are mainly vertically transmitted from the mother and placenta-derived tissues also play a significant role in the formation of the infant’s initial gut microbiota. Notably, none of the hereby-mentioned influences (i.e., gender, delivery mode, feeding mode, and Hepatitis B virus) affected significantly infant gut microbiota colonization.

**Conclusion:**

This study demonstrated that infant intestinal microbiota resulted from microbiotic co-provision from multiple maternal sites. In addition to the maternal gut microbiota, the placenta-derived tissues is the relevant contributor to initial infant gut microbiota, providing strong evidence for the source colonization of the infant gut microbiota.

## 1 Introduction

Human microbiota consist of trillions of bacteria. Microbial community evolution and colonization in early life is critical during both short and long-term infant development, particularly in terms of immunity (Kau et al., 2011), metabolism (Wu et al., 2020), and neurodevelopment (Wu et al., 2020; Ahrens et al., 2024). Microbiota could reportedly affect human health, and diseases that are more prevalent in infancy (e.g., necrotizing colitis, Crohn’s disease, type 1 diabetes mellitus, and asthma), being among the key focus areas of current disease prevention research. Microbial community establishment during early infancy is imperfect and susceptible to various factors. The delivery (Kim et al., 2019; Mitchell et al., 2020) and feeding (Kim et al., 2019; Inchingolo et al., 2024) modes, maternal diet (Catassi et al., 2024), and pharmacological interventions (Nelson, 2022) reportedly affect infant gut microbial community colonization directly or indirectly. Different influencing factors have been assessed in detailed studies. Delivery significantly affects gut microbial colonization (Shao et al., 2019; Kim et al., 2020; Yang R. et al., 2024). An important microbiotic source of the infant gut is the maternal vaginal microbiota. Cesarean section-born and naturally delivered infants carry significantly different microbiota (Dominguez-Bello et al., 2016): as the former do not pass through the maternal vagina, they potentially lack certain microbiota in early life that might affect early-life microbiotic diversity and stability (Stinson et al., 2018; Baud et al., 2023; Jaspan et al., 2023). Feeding practices also influence gut microbiotic composition. Breast- and formula feeding affect microbiota colonization, with the latter yielding reduced microbial diversity (Ruan et al., 2023).

Before the sterile uterus concept was debunked, unborn children were assumed to develop in a sterile environment, and the external environment, to which the infant was exposed during birth through the delivery mode, feeding practices (Zhang et al., 2019; Ma et al., 2023), the hospital environment, and the first contacted people, was hypothesized to shape the early-life infant microbiota. However, the sterile uterus concept has been increasingly challenged and slowly disintegrated by subsequent results. Aagaard et al. (2014) revealed that endemic microbiota in the placenta exhibits low abundance and metabolic richness. Paul et al. (2016) proved in-utero maternal microbiota transmission in animal models. Baker et al. (2018) discovered uterine microbiota. Stinson et al. (2019) obtained bacterial DNA from amniotic fluid tests and first-expelled meconium, suggesting that in utero infants display microbiota. Hence, the sterile uterus concept does not stand firmly any longer. Subsequent studies provided further evidence supporting the concept of infant gut microbiota colonizing from the uterus. However, considerable controversy remained over the subject and subsequent studies yielded different results. In 2019, Kuperman et al. did not detect any bacteria within the placenta and claimed that it contained only extremely minute microbiotic amounts, if any (Kuperman et al., 2020). In 2021, the results of Turunen et al. on placental and amniotic fluid microbiota did not support the presence of placental and amniotic fluid microbiota in healthy pregnancies (Turunen et al., 2021).

Early-life gut microbiota development affects long-term health (Nelson, 2022). Therefore, exploring infant gut microbe colonization sources is critical, with considerable differences remaining between studies on this aspect. A comparative microbial community study between infants in the first month of their lives and their mothers demonstrated that microbial communities of the maternal and infant skin are the most similar, followed by those of the feces, saliva, and the nasopharynx (Ferretti et al., 2018a). Other studies presented that amniotic fluid microbiota contributed the most to seeding the meconium microbiota (He et al., 2020). Furthermore, several studies addressed the vertical *Bifidobacterium bifidum* transmission in the mother (Duranti et al., 2017; Selma-Royo et al., 2024) and that of the mother–infant microbiota also contributes to infant gut microbiota colonization (Murphy et al., 2023), potentially as each experiment relied on sample collection and manipulation methods retaining a certain degree of error.

The Hepatitis B virus (HBV) might be present in the blood, saliva, breast milk, sweat, tears, nasopharyngeal secretions, semen, and vaginal secretions. HBV vertical transmission mechanisms include intrauterine infection and transmission during labor and delivery (Mavilia and Wu, 2017). Few studies investigated whether carrying HBV at birth affects microbial community colonization in early infants.

Infant gut microbiota origins have consistently been a research direction of concern. Among the results of numerous previous investigations, those on initial microbial ecology establishment in the infant gut are inconsistent. Therefore, studying infant intestinal microbiota from further perspectives is necessary to more comprehensively elucidate these phenomena.

In this study, we collected prenatal, 10–15-, and 40–45-d-postpartum maternal and infant feces as well as vaginal secretions, infant meconium, placental tissues, breast milk, amniotic membrane tissues, umbilical cord blood, and breast skin to compare their homology and explore the main infant intestinal microbiota sources. We explored the mother–infant symbiotic microbiota transmission mechanism to provide a theoretical basis for maternal nutrition intervention to achieve infant intestinal microbiota development regulation, while also considering the influence of various factors including time grouping, gender, delivery mode, feeding mode, and HBV.

## 2 Materials and methods

### 2.1 Volunteer recruitment

We carried out the volunteer recruitment through a cooperation with the Department of Obstetrics and Gynecology of Beijing Ditan Hospital affiliated with Capital Medical University, using the following recruitment criteria.

Inclusion criteria:

1. Pregnant women awaiting labor with a gestational age of 37–42 weeks;
2. Newborns with an Apgar score of > 7 and birth weight of ≥ 2,500 g;
3. Voluntary participation and signed informed consent.

Exclusion criteria:

1. Newborns screened with congenital and serious infectious diseases;
2. Parents with severe chronic diseases, i.e., HIV infection, cancer, bone marrow or organ transplantation, blood preparation input within the last 3 months bleeding disorders, or known congenital malformations or genetic defects;
3. Mothers suffering from an infectious disease other than HBV;
4. Parents or guardians who could not read or understand Chinese;
5. Families who were likely to relocate from the research area during the study period and could not complete the tracking.

We recruited a total of 33 volunteers during the implementation period, of which 5 withdrew and 2 yielded incomplete samples. We used sample data from a total of 26 volunteers for analysis, summarizing the related basic information in Table 1.

**Table 1:** Study population characteristics.

Subject	Index	Value
Mother (*n*=)	Age	31.04 ± 3.24
	Height (cm)	160.65 ± 3.94
	Delivery method	Natural birth: 17 (65.38%) Cesarean section: 9 (34.62%)
Neonate (*n*=)	Feeding methods	Breastfeeding: 20 (76.9%) Non-breastfeeding: 6 (23.08%)
	Gender	Male: 13 (50%) Female: 13 (50%)
	Height (cm)	50.31 ± 0.67
	Weight (kg)	3.52 ± 0.44
	Head circumference (cm)	34.63 ± 1.49

Of the 26 volunteers, among the mothers, 10 had HBV infection, and 16 were non-HBV controls; 17 delivered vaginally and 9 by cesarean section; 15 mothers who delivered vaginally breastfed their newborns and 2 did not; of the 9 mothers who delivered by cesarean section, 5 breastfed and 4 did not; 12 mothers were aged 26–37 years; 73% of the mothers had at least a college/Bachelor’s degree-level education; all 26 newborns were full-term infants (i.e., ≥ 37 gestational weeks), comprising 13 male and 13 female infants. The length, weight, and head circumference of all infants were within the ranges of the Chinese Reference Standards for the Growth and Development of Children Under 7 Years of Age and the World Health Organization Standards for Child Growth and Development.

### 2.2 Sample collection

We collected the samples at four phases:

1. Prenatally, we collected samples including maternal feces and vaginal secretions as well as maternal dietary records or reviews for 3 days before fecal sample collection. The participant mothers collected maternal fecal samples and completed dietary records or review forms under medical supervision. Doctors collected maternal vaginal secretion samples.
2. At the time of delivery, a medical professional collected all samples in the delivery room, including that of the umbilical cord blood, placental tissue, amniotic membrane tissue, and meconium (if at the delivery time, the first stool after birth). When collecting vaginal secretions, placental tissue, and amniotic membrane tissue from mothers in labor, the principle of aseptic operation was strictly followed, and healthcare workers sterilized their hands and wore sterile surgical gowns and gloves. We strictly sterilized all instruments used for collection, such as forceps and scissors. The operating environment was a clean and the delivery or operating room was sterile, with reduced personnel movement and air flow. When collecting placental tissue and amniotic membrane tissues, we shortened the time of exposure to the external environment as much as possible. We placed the tissue samples into a sterile container, sealed immediately after collection, and transported them to the laboratory department as soon as possible. For vaginal secretions collection, we sterilized the skin and mucous membranes around the vagina before carefully collecting the required tissue portion.
3. At 10–15 and 40–45 days postpartum, we collected maternal and infant feces samples as well as a record or review of maternal diet for the 3 days prior to fecal sampling. Moreover, for breastfeeding mothers, we collected breast milk and breast skin surface swab samples. The participant mothers conducted all sampling under professional guidance.

To ensure sample quality and the obtention of accurate results, we froze the samples at −20 °C immediately after collection in the hospital, then used ice packs to transport them to a − 80 °C refrigerator for preservation within 5 days. Mothers placed the samples they collected at home in their own refrigerators at −20 °C for freezing, and we asked them to send the samples to the hospital in 3 days.

### 2.3 16S rRNA sequencing

We collected breastmilk and umbilical cord blood samples (200–300 μL) in sterile centrifuge tubes. We cut the placental and amniotic membrane tissue samples into small pieces (approximately 2–3 mm^3^ and 0.2–0.3 g) under sterile conditions, and placed them into sterile centrifuge tubes. We placed swabs (i.e., those of the skin near the breast, and vagina) into sterile centrifuge tubes, and added to them 60 μL of the buffer provided in the kit. We vortexed the samples and oscillated them for full elution in the liquid. We isolated bacterial genomic DNA from the homogenized samples using a TIANamp Soil DNA Kit. We extracted individual samples according to the manufacturer’s instructions.

We amplified the highly variable V3–V4 region of the bacterial 16S rRNA gene for testing. The total reaction volume was 30 μL, containing 10 ng DNA template using 15 μL Phusion➅ High-Fidelity PCR Master Mix (New England Biolabs), and 0.2 μM forward and reverse primers 338F (5′-ACTCCTACGGGGAGGCAGCAG-3′) and 806R (5′-GGACTACHVGGGTWTCTAAT-3′). We carried out the first denaturation at 98 °C for 1 min, followed by 30 cycles at 98 °C (10 s), 50 °C (30 s), and 72 °C (30 s), and a final hold of 5 min at 72 °C. We subjected the constructed libraries to a ubiquitination test and quantified the results by Qubit and Q-PCR, after which we performed PE250 on-line sequencing using NovaSeq6000.

We processed the sequencing data using the QIIME2 platform, performing noise reduction using the DADA2 module of the QIIME2 software and species annotation on the aggregated data based on the Silva 138 99% OTUs full-length sequences (Bokulich et al., 2018).

### 2.4 Data analysis

We performed statistical analyses using the R statistical software (version 4.3.1). *α*-diversity (Shannon index), based on the genus level abundance profile, calculated using the Vegan package (version 2.6–8)(Oksanen et al., 2009) and compared using the Wilcoxon rank-sum test. We ran Adonis analysis of beta-diversity based on Bray–Curtis distances using the Vegan package, and performed principal co-ordinates analysis plot using the ggplot2 package (Wickham, 2016). We performed similarity analyses based on the Bray–Curtis dissimilarity between the samples.

After mother–child pairing, we performed correlation analyses using the rrcov package (Todorov and Filzmoser, 2009) using the Pearson method, calculated the mean of the *r*-values and visualized it. We generated the Venn diagram online using the Evenn website^1^ (Yang M. et al., 2024). We based our traceability analysis on SourceTracker R scripts (Knights et al., 2011). We considered differences statistically significant at *p*-values of *p* < 0.05.

## 3 Results

### 3.1 Bacterial microbiota distribution

**Figure 1:**
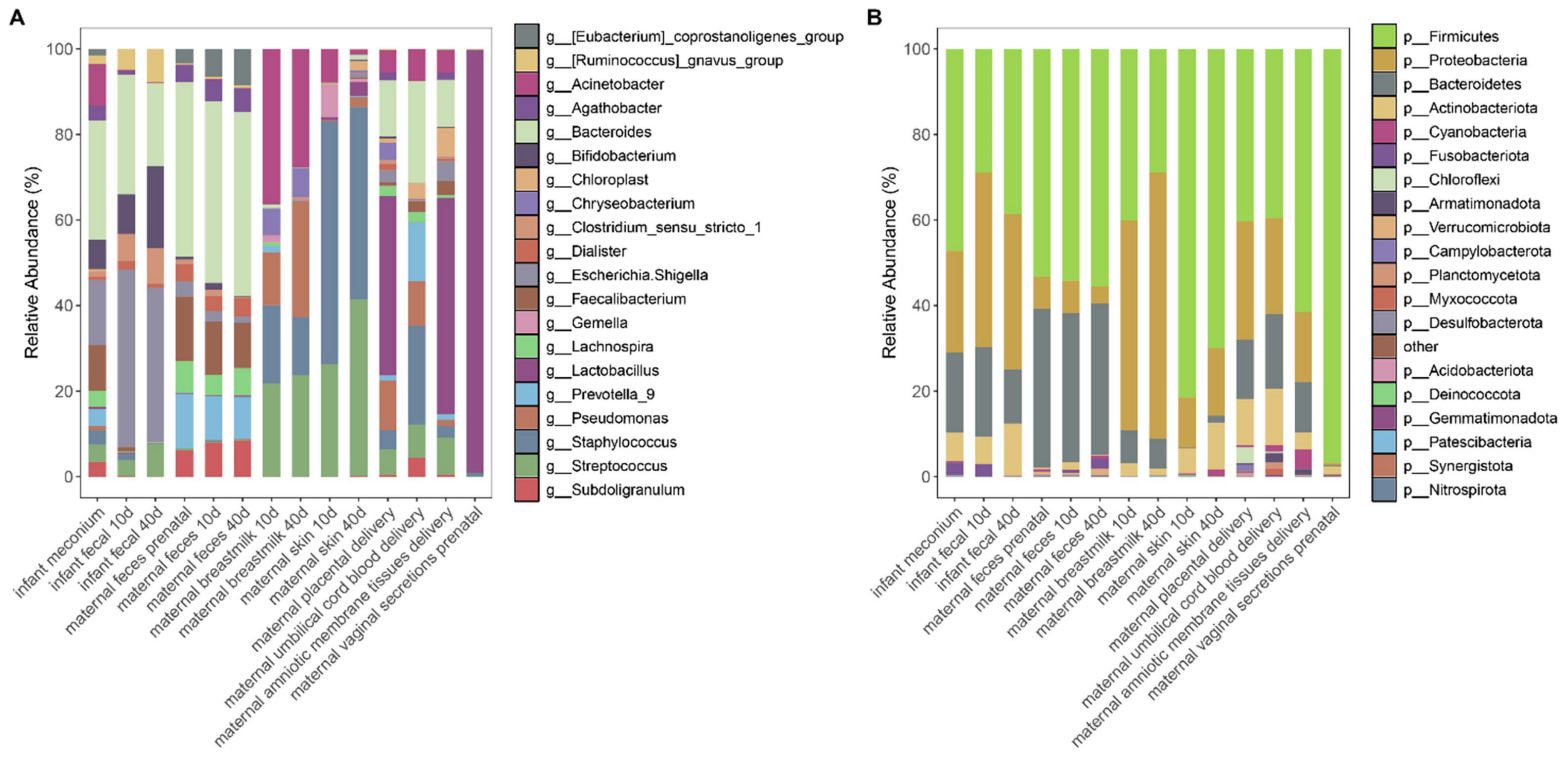
**(A)** Relative abundance of the top 20 genera in the microbial community at the genus level across all samples. **(B)** Relative abundance of the top 20 phyla in the microbial community at the phylum level across all samples.

We examined the microbiota in different maternal body habitats and maternal and infant feces at different times and constructed microbiota abundance plots (Figure 1A). The vagina, placenta, and maternal amniotic membrane tissues, in close proximity to each other, displayed similar microbial compositions, the main microbial genus being *Lactobacillus* spp. The latter was not detected in the umbilical cord blood, which in turn contained mainly *Bacteroides* and *Staphylococcus* spp. Infant meconium samples were dominated by *Bacteroides* and *Escherichia-Shigella* spp. Furthermore, we could observe that the microbiota detected in infant meconium was more abundant than that in the two later stages. *Escherichia-Shigella* spp. abundance in the 40-45-d postnatal feces of infants was lower than in the 10–15-day postnatal infant feces samples, and *Bacteroides* abundance was higher than that in the feces of 10–15-day-old infants. Maternal fecal microbiota was relatively stable in terms of species and abundance, with *Bacteroides* and *Feacalibacterium* spp. prevalent in the maternal gut. The microbial composition of breast milk remained relatively stable, with *Acinetobacter* spp. being the predominant genus, with a relative reduction in *Acinetobacter* spp. abundance and an increase in that of *Pseudomonas* spp. in 40–45-d postpartum breast milk samples compared to those from the previous phase. We observed a similar phenomenon in the microbiota of the skin near the breast, with an increased and reduced relative abundance of *Streptococcus* spp. and *Staphylococcus* spp., respectively, relative to those in the first stage. Throughout the study, the infant fecal microbiota contained the dominant genera in all samples to a greater or lesser extent.

Based on the analysis of the average microbial abundance at the phylum level, we discovered that infant fecal samples mainly comprised *Firmicutes* and *Proteobacteria*, similar to the samples of amniotic membrane tissues, the breast milk, placental, skin, and umbilical cord blood. Maternal fecal samples mainly contained *Firmicutes* and *Bacteroidetes*. Vaginal secretions was dominated by *Firmicutes* and *Actinobacteriota* (Figure 1B).

### 3.2 *α*-Diversity analyses of multiple sites

**Figure 2:**
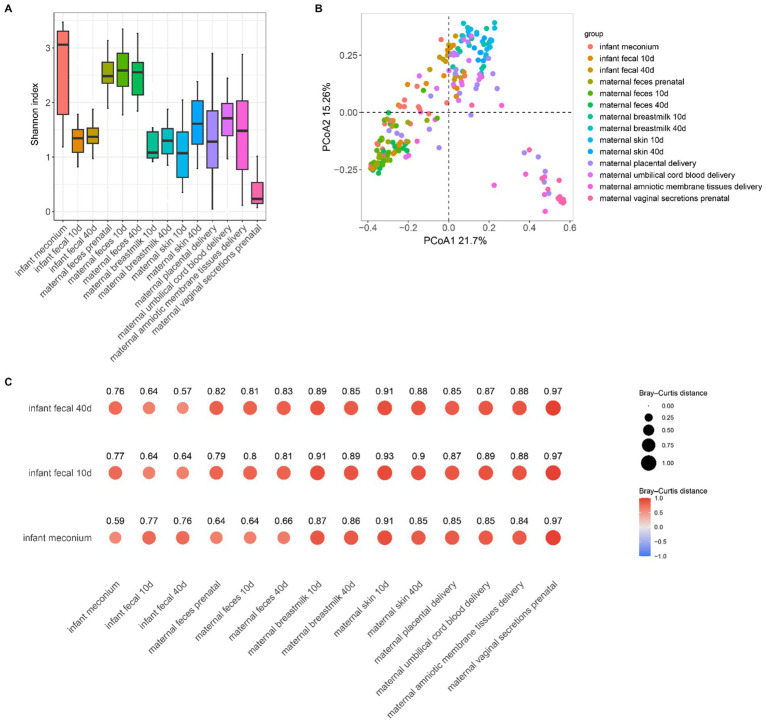
**(A)** Shannon index boxplot for each sample; **(B)** PCoA plot based on the Bray–Curtis distance for each sample. **(C)** Distance between each sampled sample based on the Bray–Curtis distance. Bubble size and color represent the relative distance values: the smaller the distance value the closer the two groups.

To further explore the source and developmental characteristics of the infant intestinal microbiota, we analyzed the α-diversity of the microbiota collected from different body parts of both mothers and infants (Figure 2A and Supplementary Table 1). Our results indicated that the α-diversity Shannon index of the infant gut microbiota was higher at birth than at the two later stages, suggesting that gut microbiota diversity was high at this time, potentially due to vertical maternal microbiota transmission. With infant development, the α-diversity Shannon index appeared to decrease, followed by a gradual increase but remaining lower than the intestinal microbiota diversity of infants at birth. Maternal fecal microbiota α-diversity was stable and almost unchanged prenatally as well as from 10–15 and 40–45 days postpartum. We detected no differences in the α-diversity of breast milk microbiota across different periods. Microbiota of the skin near the breasts displayed an increased α-diversity at 40–45 days postpartum compared to that at 10–15 days postpartum, most likely related to infant oral microbiota exposure. Maternal vaginal microbiota yielded the lowest α-diversity. Furthermore, infant meconium microbiota diversity was similar to that of the maternal intestinal microbiota, and as the infant body developed, the diversity of the gut microbiota at 10–15 days and 40–45 days is similar to that of the breast skin, breast milk, placenta, amniotic membrane tissues, and umbilical cord blood microbiota.

### 3.3 Maternal–infant microbiota *β*-diversity analyses and infant gut colonization

We performed principal coordinate analysis based on the Bray–Curtis distance (Figure 2B; Supplementary Table 2). The difference in *β*-diversity of the infant meconium and placental microbiota was not significant (*p* = 0.11), indicating that meconium microbiota was similar to maternal placenta microbiota, also suggesting that infant intestinal microbiota colonization correlated with the placental microbiota. Moreover, our results suggested that β-diversity of infant fecal microbiota in the latter two stages differed significantly from that of the microbiota of all body parts. However, this finding does not necessarily indicate that infant feces was unrelated to the microbiota of other body parts of the mother. To further investigate the correlation between the microbiota composition of the infant gut and maternal body parts, based on the Bray–Curtis distance, we calculated the distance between different samples (Figure 2C), the smaller the distance the more similar the composition of the microbiota of the two samples. We observed that the Bray–Curtis distance between the infant and maternal feces was the lowest, indicating that the microbial composition of the infant feces is more similar to that of maternal feces, demonstrating that maternal gut microbiota is dominant in infant gut microbiota colonization. However, infant feces within 10–15 and 40–45 days postpartum exhibited the smallest β-distance and high similarity of in microbiota composition, indicating minimal changes in the infant intestinal microbiota during these two postpartum phases and suggesting a stable development of the gut microbiota during this period.

In addition to the placental microbiota, the fecal microbiota of infants is also similar to that of their mothers’. Moreover, the similarity between the fecal microbiota of infants in the latter two stages and the microbiota in breast milk, as well as the skin microbiota near the mother’s breast, was significantly increased, indicating that breast milk has a certain influence on the development of infant intestinal microbiota colonization. We observed no significant difference in the maternal fecal microbiota at different sampling times.

### 3.4 Infant fecal microorganism comparison with the maternal body microbiota

**Figure 3:**
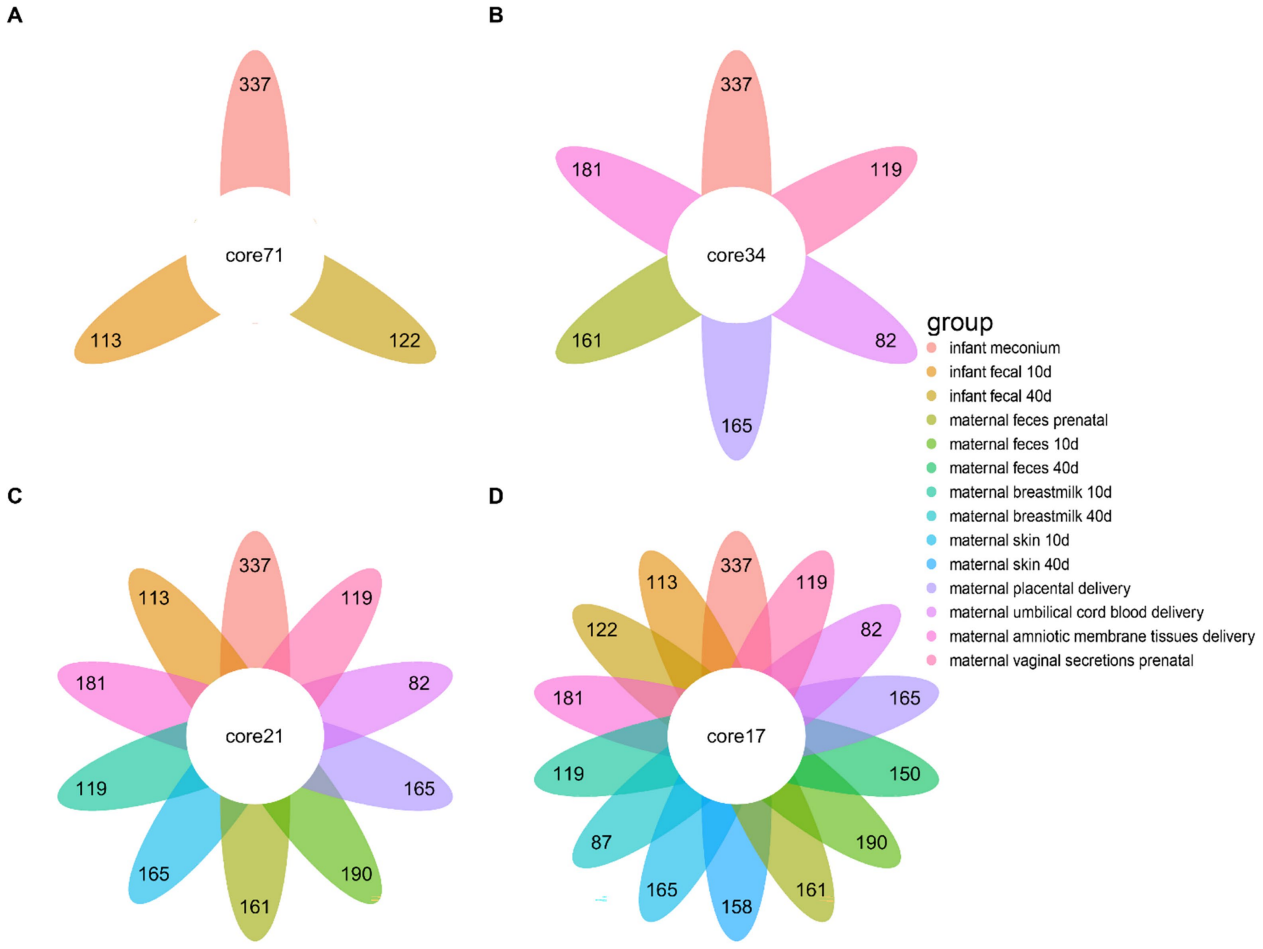
Differences in the microbial community species of infant feces at different periods compared to other bodily samples. **(A)** Differences in microbiota species of infant fecal samples at three sampling times. **(B)** Differences in microbiota composition of infant fecal samples at the time of delivery compared to other samples collected during the same period. **(C)** Differences in microbiota composition of infant fecal samples at 10–15 days postpartum compared to samples collected at the same time and in a previous period. **(D)** Difference in microbiota composition between infant feces at 40–45 days postpartum and samples taken at the same time and at previous times.

To investigate the microbial composition-related similarities between the infant fecal samples at different developmental stages and various maternal body sites, we used a Venn diagram to visualize the microbial community profiles of infant fecal samples and different parts of the bodies of the mothers (Figure 3). The number of fecal microbial genera at different stages in infants were 122, 113, and 337, and the 3 stages contained 71 common microbial genera (Figure 3A). The infant meconium and maternal amniotic membrane tissue, prenatal feces, placenta, umbilical cord blood, and vaginal microbiota sample comparisons revealed common microbial genus numbers of 337, 181, 161, 165, 82, and 119, respectively, with 6 sites containing 34 common microbial genera (Figure 3B). The comparison of infant feces at the 10–15-day postpartum stage with different body parts at the same stage and at the first stage indicated 21 common microbial genera. Comparing infant feces at the 40–45-day postpartum stage with different body parts at the same stage and the first two stages revealed 17 common microbial genera (Figures 3C,D), indicating the existence of certain non-persistent bacteria during the initial colonization period that gradually disappeared with infant development. The remaining persistent bacteria played an important role in infant intestinal microbiota.

### 3.5 Maternal and infant gut microbiota source trace analysis

**Figure 4:**
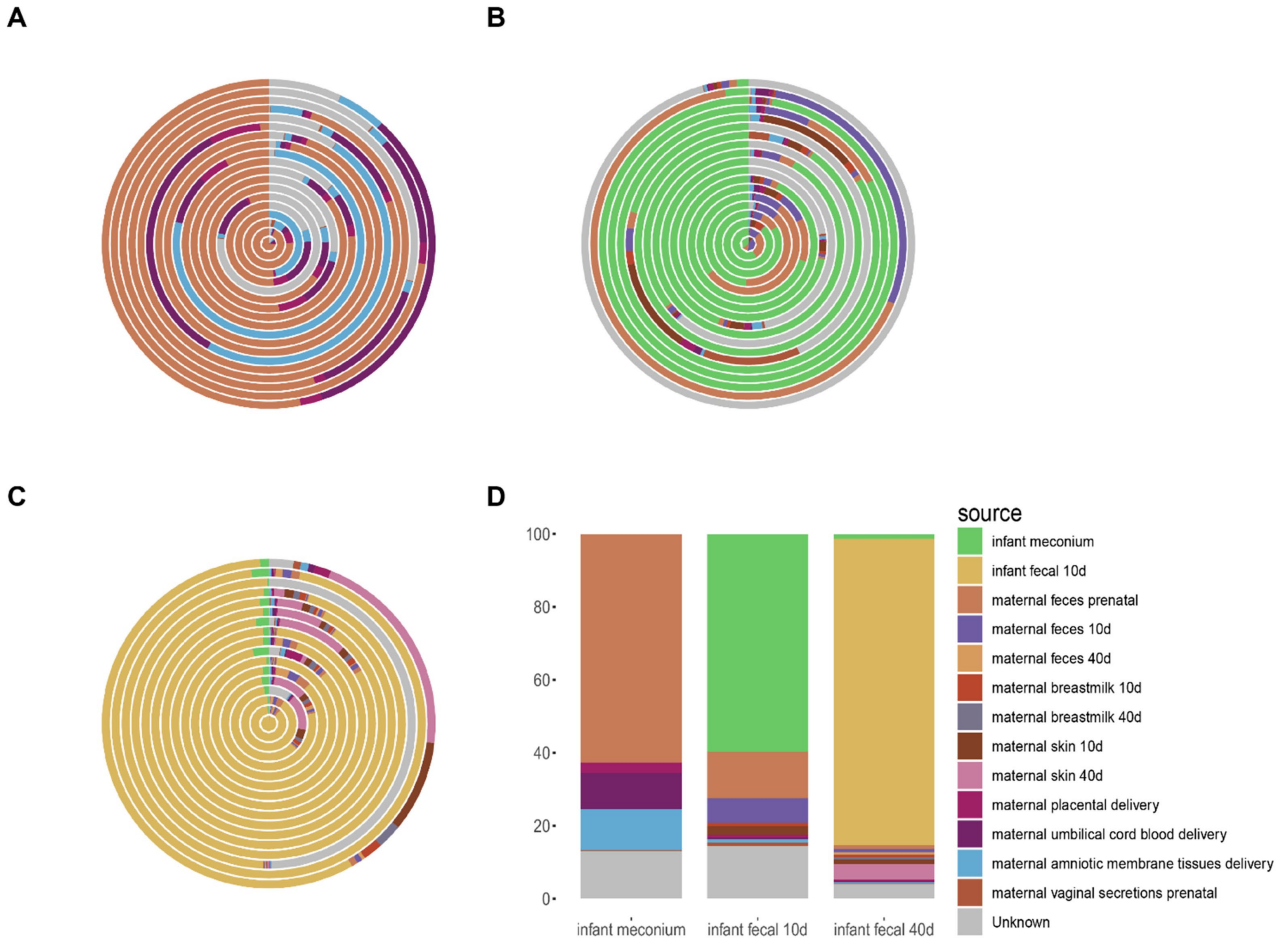
Source tracking analysis of fecal microbiota in infants. **(A)** Source tracking results of microbial communities in individual infant fecal samples at delivery, compared with other samples collected during the same period. **(B)** Source tracking results of microbial communities in individual infant fecal samples from 0 to 15 days postpartum, compared with samples collected during the same period and earlier time points. **(C)** Source tracking results of microbial communities in individual infant fecal samples from 40 to 45 days postpartum, compared with samples collected during the same period and earlier time points. **(D)** Average source tracking results of fecal microbiota samples across different time points. **(A–C)** Each ring in the figure represents the source tracking result of an individual infant fecal sample.

Next, we traced infant gut microbiota at three stages based on other sites. Infant meconium microbiota mainly derived from maternal prenatal fecal microbiota, and to a lesser extent from maternal amniotic membrane tissue, placenta, and umbilical cord blood microbiota (Figure 4A). Infant intestinal microbiota at the 10–15-day postpartum stage mainly derived from the own intestinal microbiota of the infant inherited from the previous stage as well as from the maternal prenatal fecal microbiota (Figure 4B). Infant intestinal microbiota at the 40–45-day postpartum stage mainly derived from infant fecal microbiota at the 10–15-day postpartum stage (Figure 4C). As observed in the average microbiota source trace (Figure 4D), the fecal microbiota of infants at 10–15 days and 40–45 days primarily originates from the inheritance of gut microbiota from the previous stage. The infant gut microbiota mainly derives from the maternal prenatal fecal microbiota, with smaller contributions from the maternal amniotic tissue, placenta, and umbilical cord blood microbiota.

### 3.6 Infant and maternal microbial correlations

**Figure 5:**
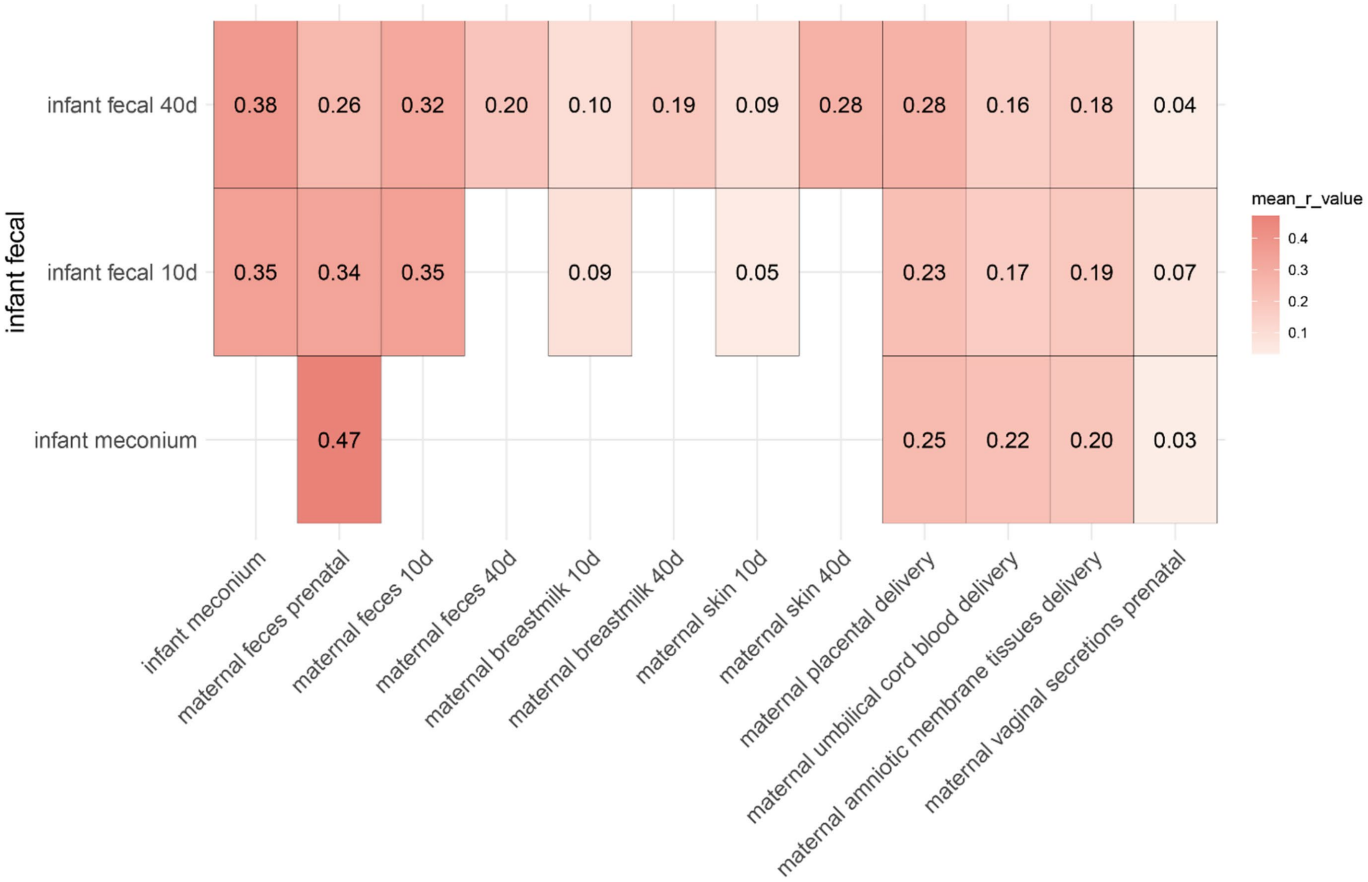
Mean correlation between infant feces and other samples at different sampling times.

The microorganism composition of the meconium was similar to that of the maternal prenatal gut microbiota as well as that of the umbilical cord blood, placenta, and amniotic membrane tissues at delivery. Therefore, we performed a correlation analysis between infant feces and the maternal prenatal gut microbiota as well as those in umbilical cord blood, placenta, and amniotic membrane tissues at delivery (Figure 5).

The correlation analysis of the infant fecal microbiota with the microbiota in different body parts of the mother revealed that infant meconium gut microbes had a similar composition to the maternal prenatal gut microbiota, placental microbiota, umbilical cord blood microbiota, and amniotic membrane tissue microbiota, with significant correlation coefficients of 0.47, 0.25, 0.22, and 0.20, respectively. Maternal prenatal fecal microbiota correlated with the microbiota of the infant meconium. Vaginal microbiota did not correlate with the infant intestinal microbiota.

At 10–15 days, infant fecal microbiota was associated with infant meconium (*r* = 0.35), maternal feces 10 days (*r* = 0.35), maternal feces prenatal (*r* = 0.34), placenta (*r* = 0.23), umbilical cord blood (*r* = 0.17), and amniotic membrane tissue (*r* = 0.19). At 40–45 days, infant fecal microbiota was associated with infant meconium (*r* = 0.38), maternal feces 10 days (*r* = 0.32), maternal feces prenatal (*r* = 0.26), maternal feces 40 days (*r* = 0.2), maternal breastmilk 40 days (*r* = 0.19), maternal skin 40 days (*r* = 0.28), placenta (*r* = 0.28), umbilical cord blood (*r* = 0.16), and amniotic membrane tissue (*r* = 0.18).

### 3.7 Infant gut microbiota-affecting factors

Multivariate analysis of variance (Adonis) detected no statistically significant difference in the effect of time grouping (*p* = 0.216), gender (*p* = 0.635), delivery mode (*p* = 0.136), feeding mode (*p* = 0.831), or HBV (*p* = 0.335) on infant gut microbiota colonization.

## 4 Discussion

Microbial diversity differs among different bodily locations. Although each site has its own microbial composition, these communities interact (Ferretti et al., 2018a). Microbiota could be both beneficial and harmful to the host. Several studies demonstrated that the gut microbiota is associated with various diseases including intestinal disorders (Fehily et al., 2021) as well as celiac (Marasco et al., 2016), renal (Xie et al., 2018), and liver (Jones and Neish, 2021) diseases, or autoimmune and metabolic disorders (Woting and Blaut, 2016; Xu et al., 2019), affecting infant brain development (Wang et al., 2018). Hence, exploring infant gut microbiota sources is crucial (Goulet, 2015). The main initial gut microbiota establishment source in infants is vertical microbiotic transmission from the mother (Yassour et al., 2018). However, no uniformity exists concerning the main contributors to the infant gut microbiota, with certain studies suggesting amniotic fluid as the most important contributor, which shapes a comfortable and viable living and developmental environment for infants within the body of the mother (He et al., 2020), and others positioning the maternal gut microbiota as the main contributor (Sakwinska et al., 2017; Turunen et al., 2021).

In this study, we investigated the origin of infant gut microbiota, and analyzed microbial genera, abundance, diversity, and source trace in infant feces and at various habitats in the mother. We analyzed the microbiotic genera and their abundance in different body parts both of the mother and infant at different times. Our results were similar to the conclusions previous studies drew (Williams et al., 2019), with the predominant genus being *Bacteroides* in the feces, the skin tissues in the vicinity of the breasts being dominated by *Staphylococcus* spp. and *Streptococcus* spp., and breast milk by *Acinetobacter*.

*α*-diversity revealed that the microbial species and abundance in fecal samples, as well as in skin and breastmilk samples near the breast, all changed across the three maternal and infant sampling stages (at delivery, 10–15 days postpartum, and 40–45 days postpartum). α-diversity alterations of the maternal feces was not obvious as the maternal intestinal microbiota was a relatively mature microbial community, and the α-diversity of the skin microbiota near the breasts increased at 40–45 d postpartum, potentially as a result of interactions between the maternal and infant microbiota. In good agreement with previous findings (Ferretti et al., 2018a), the α-diversity of the microbial community at the time of infant delivery was markedly higher than that of the maternal fecal microbiota during the latter two stages, suggesting that the initial stages of infant intestinal microbial species are relevantly more diverse than we perceive and intestinal microbiota colonization is not due to any one site. Moreover, maternal site microbiota reportedly contributes to infant gut microbiota establishment (Ferretti et al., 2018b). A normal gut microbiota contributes to gut function development from the time the infant is born. Mothers are closely associated with the microbiota of their infants by helping to establish it through their own gut microbiota, *in utero*, birth canal environments, and breastfeeding (Yang et al., 2021). With infant development, the α-diversity Shannon index appeared to decrease, possibly due to the environmental changes affecting the dominant intestinal bacteria, resulting in the reduction (or disappearance) of those that were not adapted to the new environment, consistent with previous studies (Adlerberth, 2008; Ferretti et al., 2018b; Stewart et al., 2018).

In this study, we detected microbiota in the placenta, amniotic membrane tissue, and umbilical cord blood, supporting the idea that the uterus is not sterile. Our *β*-diversity results revealed that the distance between the initial intestinal microbiota of the infant and the placental microbiota was shorter than that to the vaginal microbiota, most likely due to the placenta being the main material exchange site between the infant and mother, through which the fetus receives substances such as oxygen, nutrients, and hormones from the mother, while expelling carbon dioxide and other fetal wastes (Al-Enazy et al., 2017). During infant development, oxygen in the gut of the infant is gradually consumed by aerobic bacteria, and the reduced oxygen content allows for anaerobic bacteria to grow and colonize the gut (Albenberg et al., 2014), representing an important modification in the gradual development of infant intestinal microbiota toward maturity. As the infant grows and develops, infant intestinal microbiota in the two later stages more closely resembles those of the breast milk microbiota, potentially as a result of the continuous development of the infant intestinal microbiota and breastfeeding acceptance (Koren et al., 2012).

This study described the microbial communities in maternal amniotic membrane tissues, placenta, and umbilical cord blood, which is consistent with previous results stating that identified bacteria in the placenta, umbilical cord blood, and amniotic membrane tissues (Aagaard et al., 2014; Carmen Collado et al., 2016; Yin et al., 2023). And in this study, the microbial communities in maternal amniotic membrane tissues, placenta, and umbilical cord blood showed no significant β-diversity. These results suggested that the fetus might not be completely sterile *in utero*, and that initial colonization of the fetal intestinal microbiota may indeed begin in the maternal uterus. In this study, we observed that vaginal microbes did not affect infant gut microbes, consistent with the findings of Dos Santos et al. (2023) that vaginal microbes do not affect infant gut microbes.

The retrospective results demonstrated that infant intestinal microbiota was mainly due to vertical transmission of maternal intestinal microbiota and inheritance of infant fecal microbiota from the previous stage, in line with the results of most previous reports, and that maternal intestinal microbiota retains the most obvious and long-lasting influence on infant intestinal microbiota (Asnicar et al., 2017; Maqsood et al., 2019). In addition, placenta-derived tissues also have a significant impact on infant gut colonization.

Our correlation analysis indicated that the meconium strongly correlated with maternal prenatal intestinal microbiota and weakly with that in the umbilical cord blood, placenta, and amniotic membrane tissue. The fetus is connected to the mother through the umbilical cord, and umbilical blood circulation delivers oxygen and nutrients to the fetus. The high abundance in the microbiota composition in umbilical cord blood and meconium strongly indicated that the microbiota in the umbilical cord blood delivered by the mother to the fetus originated from the mother. Blood is composed of two parts (i.e., plasma and blood cells) and nutrients in the plasma are mainly absorbed through the intestinal system. Hence, we could preliminarily hypothesize that microbiota in the umbilical cord blood might derive from maternal intestinal microbiota. The fetus exchanges substances with the mother through the placenta, and the amniotic tissue is a membrane on the inside of the placenta, indirectly exchanging substances (including microbiota) with the maternal blood. Based on this result, we hypothesized that the infant intestinal microbiota originated mainly from the mother through vertical transmission.

Initial gut microbial colonization is influenced by multiple factors (Vandenplas et al., 2020). The delivery mode reportedly affects infant intestinal microbiota for up to 6 months, 1 year, and 7 years of age (Neu and Rushing, 2011; Chu et al., 2017; Shao et al., 2019). However, in this study, multivariate variance indicated that the time grouping, gender, delivery mode, feeding mode, and HBV did not significantly affect infant intestinal microbiota colonization. This suggests that the colonization of an infant’s gut microbiota may be primarily influenced by vertical maternal transmission rather than external environmental factors. Ahn et al. (2023) analyzed the fecal microbiota and metabolites of the entire gastrointestinal tract and concluded that fecal microbiota did not sufficiently represent the entire intestinal microbiota. This study assessed the gut microbiota solely based on infant fecal samples, which may not fully represent the microbial composition of the entire gastrointestinal tract. The 16S rRNA sequencing method used in this study limited the resolution of the samples to the genus level, subsequent related studies could incorporate macro genomics for more detailed experiments.

## 5 Conclusion

By analyzing the bacterial composition, *α*- and *β*-diversity, and source trace of 26 mother–infant samples, we demonstrated in this study that infant intestinal microbiota was the result of microbiota co-provision from multiple sites of the mother. In addition to the maternal gut microbiota, placenta-derived tissues also play a significant role in the formation of the infant’s initial gut microbiota. Non-persistent bacteria in the infant intestinal microbiota decreased or disappeared during later development, with persistent bacteria remaining. Subsequently, infant intestinal microbiota was more closely aligned with that of the mother as a result of vertical transmission from the mother and the tendency of the infant intestinal microbiota to stabilize its development. In summary, this study provides a basis to reveal the source of microbiota in the infant gut and exploring the transmission mechanism of symbiotic microbiota between mothers and infants.

## Data Availability

The data presented in the study are deposited in the National Center for Biotechnology Information (NCBI) repository, accession number PRJNA1243050.

## Footnotes

^1^ http://www.ehbio.com/test/venn/#/
